# Supplementary material for: Healthy Behaviors through Behavioral Design–Obesity Prevention
Source: Int J Environ Res Public Health. 2020 Jul 14;17(14):5049. doi: 10.3390/ijerph17145049 (PMC7400269; doi:10.3390/ijerph17145049)
Supplement: Supplementary file 1 [file ijerph-17-05049-s001.pdf]

Table S1: Nutrition Interventions for the Built Environment for Behavior Modification

Author (year)	Design	Setting (# Sites)	Sample	Intervention / Variables	Duration	Outcomes		Summarized findings	
						Primary	Secondary	Primary	Secondary
Arsenault, Singleton, and Funderburk 2014	Nonrandomized, controlled study	Military dining facilities (5)	n = 299	Environmental. Food labeling	2 months	Nutrient intake of food label users vs. Non-users	Supplement use of food label users vs. Non-users	Nutrient intake was lower for label users	Label users more likely to take supplements
Cole et al. 2018	Nonrandomized, controlled time series study	Military dining facilities (2)	n = 573	Environmental. Food labeling; choice architecture; menu modification	12 months	Nutrient intake	Healthy Eating Index (HEI) 2010 scores	Improved food selection	Higher posttest HEI score
Crombie et al. 2013	Randomized, controlled, partial crossover	Military dining facilities (10)	n = 1579	Environmental. Modifying food service operations	12 months	Mealtime nutritional intake	N/A	Significant reduction of intake of energy	N/A
Geaney et al. 2016	Cluster controlled trial	Manufacturing workplaces (4)	n = 850	Multicomponent. Nutrition education; environmental modification	9 months	Nutrient intake	BMI	Significant improvement in food selection.	Small but significant reduction in BMI
Hua et al. 2017	Randomized Factorial Trial	University Vending, U.S. (1)	n = 112	Environmental. Food labeling; choice architecture; monetary incentive	10 months	Units sold	N/A	Units sold increased	N/A

Kongsbak et al. 2016	Controlled trial	FoodScape Laboratory (1)	n = 65	Choice architecture	1 day	Fruit and vegetable consumption	Total energy	Fruit and vegetable consumption increased	Total energy decreased
Levy et al. 2012	Two-phase point-of-purchase intervention	Worksite cafeterias, (1)	n = 4642	Environmental. Food labeling; choice architecture	9 months	Item Sales	N/A	Food and beverage choices were improved	N/A
Lowe et al., 2010	Randomized trial	Worksite cafeterias, (1)	n= 96	Multicomponent health education. Healthy options increased, labeled and reduced in price	6 months	Calorie content of purchased foods	Weight change	Energy and percent of energy from fat decreased	No significant difference in weight
Nyberg & Doktor Olsen, 2010	Review paper	N/A	Two studies	Environmental. Effect of built environment on meal	N/A	Meal Satisfaction	N/A	Architectural and social consideration s should be made when planning healthy environment	N/A
Pharis, Colby, Wagner, & Mallya, 2018	Pretest-posttest	City Vending U.S., (1)	n= 250	Environmental. Increase in healthy options, positioning, and price incentives for healthy options in vending machines	4 years	Item Sales	N/A	Healthy sales increased, but overall sales decreased	N/A

Rosi et al., 2017	Pretest-posttest	University Vending Italy, (1)	n= 3	Environmental. Increase of healthy option availability and healthy option promotion through point of sale nutrition information	21 months	Item Sales	N/A	Poor food choices were discouraged, but selection of healthy option did not increase	N/A
Thorndike et al. 2012	2-phase intervention	Worksite cafeterias, (1)	N/A	Environmental. Food labeling; choice architecture	6 months	Item Sales	N/A	Sale of healthy items was enhanced	N/A
Thorndike et al. 2014	Longitudinal pre–post cohort follow-up study	Worksite cafeterias, (1)	n = 7431	Environmental. Food labeling; choice architecture	24 months	Item Sales	N/A	Sustained healthier choices over two years	N/A

Table S2: Physical Activity Interventions for the Built Environment for Behavior Modification

**Author (year)**	**Design**	**Setting (# Sites)**	**Sample**	**Intervention / Variables**	**Duration**	**Outcomes**		**Summarized findings**	
						**Primary**	**Secondary**	**Primary**	**Secondary**
Adlakha et al., 2015	Systematic Review	Residential and Workplace	N=2,015	Environmental (i.e. availability of fruits and vegetables, presence of shops and stores, bike facilities, recreation facilities, crime rate, seeing others active, and interesting things)	2012-2013	Physical activity level	N/A	Diverse, attractive, and walkable neighborhoods around workplaces increase physical activity level	N/A

Bellicha et al., 2015	Systematic Review	Worksite & Public Settings	N=60 studies	Walking Interventions (i.e. directional and motivational prompts)	N/A	Total stair use	N/A	Stair use increased and remained elevated post-intervention period	N/A
Brown et al., 2012	Randomized Control Trial	Office Worksite	N=73	Nature & Built Walking Routes	8 wks.	Resting Autonomic Function (HRV & HR) Physical Activity	Mental Health	Both walking conditions increased physical activity but did not affect resting autonomic function	Nature walking routes increased self-reported mental health
Jennings et al., 2017	Systematic Review	Worksite & Public Settings	N=67 studies	Walking Interventions (compared to elevators, escalators, or moving stairways)	N/A	Total stair use	N/A	Stair use increased in public setting (less supported in worksites)	N/A
Laine et al., 2014	Systematic Review	N/A	N=10 studies	Bicycle and Pedestrian Trails	N/A	Physical Activity [Metabolic Equivalent Tasks (MET)-hours per person per day]	Cost Efficiency	Bicycle and pedestrian trails were the most effective at increasing physical activity	Rail Trails, pedometers, and school health education programs were the most cost efficient

Leavey & Jancey, 2016	Qualitative	Office Worksite	N= 36	Sit-to-stand workstations	4 wks.	Sitting Time	General Well Being Workability Staff Engagement Mood	Sit-to-stand workstations decreased prolonged sitting time	Sit-to-stand workstations improved mood, general wellbeing, workability, and staff engagement
Myers et al., 2010	Longitudinal experimental	Hospital	N=69	Prompts (i.e. posters and floor stickers) at the point of choice between stairs and elevators	12 wks.	Total stair use VO2max Waist circumference e Weight Fat mass Diastolic blood pressure Low-density lipoprotein cholesterol	N/A	Use of prompts increases stair use and effectively improves fitness, body composition, blood pressure, and lipid profile in inactive individuals	N/A
Mutrie et al., 2002	Randomized Control Trial	Worksite	N=295	"Walk In to Work Out" packet (containing walking/cycling routes, health, and safety information)	6 mo	Walking Time	N/A	Information-based walking intervention significantly increased walking time compared to control	N/A

Neuhaus et al., 2014	Systematic Review; Meta-analysis	Office Worksite	N=38 studies	Activity-permissive workstations	N/A	Sedentary Time	Work Performance Health Outcomes	Activity-permissive workstations decreased sedentary time	Activity-permissive workstations do not affect work performance and more research is needed to determine its effects on health outcomes
Neuhaus et al., 2014	Randomized Control Trial	Office Worksite	N=44	Sit-to-stand workstations	6 mo	Sitting Time	N/A	Sit-to-stand workstations decreased workplace sitting time	N/A
Olander & Eves, 2011	Observational	Office Worksite	N=803	Elevator Availability Building Occupancy Time of Day Pedestrian Traffic	24 non-consecutive days	Total stair use	N/A	3 versus 4 available elevators, increased building occupancy, earlier time of day and decreased pedestrian traffic increase stair usage in the worksite	N/A

Puig-Ribera et al., 2008	Randomized Control Trial	University	N=70	Walking in the Workplace	9 wks.	Step Counts Well Being Work Performance Quality of Life	N/A	Sedentary participants increased step count, quality of life, well-being, and work productivity	N/A
Ruff et al., 2014	Qualitative (Survey)	Urban Worksite	N=1,348	Stair Prompts; Naturally lit stairwells; Stairwell Visibility	N/A	Total stair use; Stair use w/ stairwell visibility and natural light	N/A	Use of prompts and increases in stairwell availability and natural light can increase stair usage	N/A
Sallis et al., 2008	Systematic Review	N/A	N=7 studies	Environmental & Policy Variables	N/A	Physical Activity	N/A	Stair prompts are effective at increasing stair use while more research is needed for other variables	N/A

Soler et al., 2010	Systematic Review	N/A	N=16 studies	Point-of-decision prompts; stairwell enhancement + point-of-decision prompts	N/A	Total stair use	N/A	Point-of- decision prompts appear to be effective in increasing stair use	N/A
Thøgersen Ntoumani et al., 2014	Uncontrolled Experimental Design	University	N=75	Lunchtime Walking	16 wks.	Perceptions of Health Subjective Vitality Work Performance Fatigue	N/A	Lunchtime walking increases in perceptions of health, subjective vitality, and work performance, and decreases in fatigue at work	N/A
Van Nieuw-Amerongen et al., 2011	Observational	University	N=21,786	Stair Prompts; Stairwell Accessibility, Visibility, and Aesthetics	7 wks.	Total stair use (immediately post- and 4-wks post-intervention)	N/A	Use of prompts and increased attractiveness, visibility, and accessibility of the stairwell increases stair usage	N/A

Table S3: Indoor Environmental Quality (IEQ) Interventions for the Built Environment for Behavior Modification

**Author (year)**	**Design**	**Setting (# Sites)**	**Sample**	**Intervention / Variables**	**Duration**	**Outcomes**		**Summarized findings**	
						**Primary**	**Secondary**	**Primary**	**Secondary**

An et al., 2016	Survey	N/A	n= 444	Natural elements and direct and indirect sunlight exposure	N/A	Employee mental health and work attitudes	N/A	Natural elements and sunlight exposure have positive effects on employee mental health and job attitudes	N/A
Anothaisintawee et al., 2016	Meta-analysis	N/A	36 studies	Comparison of sleep disturbance to traditional risk factors	N/A	Diabetes risk	N/A	Sleep disturbances are significant risk factors similar to traditional risk factors	Sleep disturbances should be considered in screening processes
Bernhofer et al., 2014	Descriptive correlational	Hospital, 1	n= 40	Light exposure and sleep wake patterns	11 mo	Mood, pain, fatigue, and relationships	N/A	Fatigue and pain positively and highly correlated and low light exposure	N/A
Beute, F., de Kort,	Survey	N/A	n=59	Nature and daylight	6 days	Mood, stress, energy,	N/A	Nature and daylight were	N/A

Y. A. W., 2018						and activity		beneficial to mental wellbeing	
Boubekri et al., 2014	Observational	Office, 2	n= 49	Windows, windowless environments, and daylight exposure	2 weeks	Sleep quality, physical activity, and subjective wellbeing	N/A	More light exposure trended towards more physical activity, longer sleep duration, and better quality of life rating	N/A
Cho et al., 2015	Literature review	N/A	85 studies	Artificial light at night exposure (ALAN)	N/A	Circadian disruption	Negative health effects	ALAN causes circadian phase disruption	Circadian disruption may have negative effects on psychological, cardiovascular, and metabolic functions
Daugaard et al., 2019	Observational	Various employers	n= 509	Light exposure among indoor, outdoor and night workers	7 days	Light intensity	Overall wellbeing	Night workers exposed to exposed to	Outdoor workers exposed to light

								light intensity expected to suppress melatonin, indoor workers light exposure expected to reduce overall wellbeing	comparable treatments for depression
Erren et al., 2008	Meta-analysis	N/A	21 studies	Flight personnel and shift workers chrono disruption	N/A	Breast and prostate cancer risks	N/A	Chronodisruption may have increased breast and prostate cancer risks	N/A
Figueiro et al., 2017	Observational	Office, 5	n= 109	Circadian effective light exposure	7 days	Mood and sleep	N/A	High levels of morning circadian-effective light exposure associated with reduced sleep onset latency,	High levels of circadian-effective light associated with increased phasor magnitudes, reduced depression, and

								increased circadian entertainment and increased sleep quality	increased sleep quality
Gan et al., 2015	Meta-analysis	N/A	12 studies	Effect of shift work	N/A	Risk of diabetes mellitus	N/A	Shift work associated with increased risk of diabetes mellitus	N/A
Hadi et al., 2016	Survey	Hospital, 1	n= 393	Lighting environment for nurses	N/A	Lighting perceptions	Satisfaction, amount of lighting, disturbing conditions, controls, importane of controls and ease of controls	Relationship found between nurses' access to lighting controls and satisfaction about the lighting environment	Lighting conditions at patient besides and decentralized nurse stations less desirable than other nurse locations
He et al., 2015	Meta-analysis	N/A	28 studies	Circadian disruption and associated causes	N/A	Breast cancer risk	N/A	Positive association between circadian disruption and breast	N/A

								cancer risk in women	
Hoffmann et al., 2008	Crossover experimental	Office, 2	n= 11	Variable lighting conditions and circadian parameters	3 days	Urinary sulphatoxymelatonin (aMT6-s) and neopterin	Subjective mood	Potential benefit of variable lighting in indoor offices with respect to subjective mood and activation	N/A
Jia et al., 2013	Meta-analysis	N/A	13 studies	Effect of night work	N/A	Risk of breast cancer	N/A	Night work is associated with increased risk of breast cancer.	N/A
Joines et al., 2015	Randomized control trial	Office, 1	n= 95	Benefits of adjustable LED task lighting	6 mo	Ergonomic and calculated utility power consumption	Eye fatigue, perception of job content, intervention usability, and musculoskeletal discomfort	Benefits to musculoskeletal comfort, posture, and visual comfort with adjustable task light use	Positive assessment of light usability, usefulness, and desirability

Kamdar et al., 2013	Meta-analysis	N/A	15 studies	Effect of night shift work	N/A	Breast cancer risk	N/A	Weak evidence to suggest night-shift work is associated with increased breast cancer risk	N/A
Kazemi et al., 2018	Experimental	Confined room, 1	n= 20	Effects of various light sources	2 mo	Task performance, visual comfort and alertness	N/A	Performance was better with exposure to cold color temperature	Performance is affected by color temperature not lamp type
Lee et al., 2017	Meta-analysis	N/A	11 studies	Effect of night shift work	N/A	Risk of depression	N/A	Night shift work associated with the increased risk of depression	
Lin et al., 2015	Meta-analysis	N/A	16 studies	Effect of night shift work	N/A	Breast cancer morbidity	All-cause mortality	Night shift work associated with an increased risk of breast	Night shift work associated with an increased risk of all-

								cancer morbidity	cause mortality
Megdal et al., 2015	Meta-analysis	N/A	13 studies	Effect of night shift work and working as a flight attendant	N/A	Risk of breast cancer	N/A	Night shift work increases the risk of breast cancer	Work as a flight attendant increases risk of breast cancer
Moreno et al., 2019	Literature review	N/A	27 studies	Effect of shift work	N/A	Negative health outcomes	N/A	Shift work linked to negative health outcomes	Lifestyle and behavioral aspects also contribute to development of disease among shift workers
Newsham et al., 2008	Cross-sectional study	Office, 9	n= 779	Effect of physical environment measurements: sound, air and temperature movement, relative humidity, concentration of air	N/A	Environment satisfaction, job satisfaction, reported importance of physical features	N/A	Specific environmental criteria are suggested to reduce risk of dissatisfaction	N/A

				pollutants and illuminance					
Nylen et al., 2014	Literature review	N/A	100+ studies	Age related changes in visual and non-visual functions among older-age workers	N/A	Light and work performan ce	N/A	Visual conditions and lighting design impact work performance in those over age 65	Ocular age-related changes and disease impact wellbeing and work performance
Park et al., 2018	Retrospectiv e cohort study	Hospital, 1	n= 85, 021	Effectiveness of natural daylight	15 years	Length of stay (LOS)	N/A	Patients with beds next to a window had shorter LOS than did those next to the door	N/A
Rao et al., 2015	Meta-analysis	N/A	8 studies	Effect of night shift work	N/A	Risk of prostate cancer	N/A	There is a positive association between night-shift work and prostate cancer risk	N/A
Sithravel et al., 2018	Experimenta l	Compute r	n= 45	Effect of dynamic	N/A	Urinary sulphatox	Subjective alertness, mood,	Supportive, dynamic lighting in	Additional 50% support on the

		laboratory,1		lighting configurations		ymelatonin (aMT6-s)	visual comfort, cognitive and visual acuity-contrast task performance	increasing oscillation resulted towards a better morning boosting effect	individuals' psychophysiological wellbeing indicators with increased oscillation compared to control constant lighting
Smolders et al., 2012	Experimental	Simulated office, 1	n= 32	The alerting and vitalizing effect of illuminance	3 mo	Subjective measures, sustained attention, and cognitive performance	N/A	Higher illuminance can improve employees' subjective feelings of alertness, vitality, and objectively measured performance.	N/A
Stocker et al., 2014	Meta-analysis	N/A	15 studies	Effect of shift work	N/A	Early reproductive outcomes of menstrual	N/A	There is a positive association between shift work and early	N/A

						cycle disruption		reproductive outcomes	
Vyas et al., 2012	Meta-analysis	N/A	34 studies	Effect of shift work	N/A	Major vascular events and disease	N/A	Shift work is associated with increased risk of vascular events and disease	N/A
Wang et al., 2013	Meta-analysis	N/A	10 studies	Effect of night shift work	N/A	Breast cancer risk	N/A	A positive dose–response relationship is present for breast cancer with increasing years of night shift	N/A
Wang et al., 2014	Meta-analysis	N/A	12 studies	Effect of night shift work	N/A	Risk of metabolic syndrome	N/A	Night shift work is significantly associated with the risk of metabolic syndrome	N/A
Wang et al., 2015	Meta-analysis	N/A	6 studies	Effect of night shift work	N/A	Colorectal cancer risk	N/A	Night shift work was	N/A

								associated with an increased risk of colorectal cancer	
Zadeh et al., 2014	Quasi-experimental	Nurses stations, 2	n= 12	Effects of windows and daylight on registered nurses	3 years	Physiological responses, sleepiness, and mood	Communication, social interaction, and frequency of human-related medication errors	May result in lowered blood pressure, increased oxygen saturation, positive effect on circadian rhythms and improved sleepiness	Increased frequency of communication and positive social interaction, no significant decrease in medical errors
